# The Role of Immunological Factors on Sudden Sensoryneural Hearing Loss

**Published:** 2018-07

**Authors:** Mohammadhossein Baradaranfar, Mohammadhossein Dadgarnia, Vahid Zand, Sedighe Vaziribozorg, Fatemeh Sadat Mirzade, Mojtaba Mirzade

**Affiliations:** 1 *Department of Otolaryngology- Head and Neck Surgery, Otorhinolaryngology Research Center, Shahid Sadoughi* * University of Medical Sciences, Yazd, Iran.*; 2 *Department of Internal Medicine, Shahid Sadoughi University of Medical Sciences, Yazd, Iran.*

**Keywords:** Antinuclear antibody, Complement C3, Complement C4, Erythrocyte sedimentation rate, *Sensorineural *hearing loss.

## Abstract

**Introduction:**

In this study, we examined the role of immunological factors on sudden sensory neural hearing loss (SSNHL).

**Materials and Methods:**

This prospective case-controlled study was performed in patients with SSNHL who referred to the ear, nose, and throat (ENT) ward. Fifty-six patients with SSNHLoccurring within a 72-hr period were selected as the case group and 56 participants who had no recent history of disease were assigned to the control group. Mean levels of immunological factors including erythrocyte sedimentation rate (ESR), C-reactive protein (CRP), rheumatoid factor (RF), antinuclear antibody (ANA), anti-neutrophil cytoplasmic antibody (ANCA) (C, P), anti-cyclic citrullinated peptide (anti-CCP), DSM, hemoglobin (Hb), complement component 3 (C3), and complement component 4 (C4) were evaluated in the blood samples in each group.

**Results:**

There were 31 male and 25 female participants in the control group, while the case group had 26 male and 30 female participants. The mean age of the participants was 36.2 ± 13.4 years in the control group and 40.80 ± 13.37 years in the case group. The two groups differed significantly in terms of mean ESR, ANA, C3, C4 and monocytes, with higher levels in the case group (P<0.05). However, differences in mean CRP, anti-Ds DNA, Hb, anti-CCP, white blood cells (WBC), neutrophils, lymphocytes, eosinophils, and platelets were not statistically significant between the case and control groups (P>0.05).

**Conclusion:**

Because some of the immunological factors investigated in this study were significantly higher in patients with SSNHL, it can be concluded that there is an association between these immunological factors and SSNHL. Further studies are recommended to accurately determine the effect of these factors on the development of SSNHL and its treatment.

## Introduction

Sudden *sensorineural* hearing loss (SSNHL) is an*urgent* medical *problemand at* the same time, one of the most controversial issues in audiology (1). Symptoms of SSNHL include unilateral or bilateral hearing loss, perineal sensation, tinnitus, dizziness, and imbalance (2-4). Because of its sudden occurrence, SSNHL can *causestress* in the affected individual,which in turn *may result in* a reduction in quality of life (5,6). SSNHL affects *5–20 *individuals*per 100,000 a*nnually (7,8). Despite extensive research on the causes of this disease, its risk factors are not yet well recognized. The incidence rate of SSNHL is increasing (9), and there is no proven or recommended treatment for the condition. Due to the various etiologies of SSNHL, its treatment has not been satisfactory (10).

In addition, because of the widespread pathology of the disease, the spontaneous improvement of affected patients, and the late referral of some patients to specialist health care and treatment services, the number of controlled studies investigating various aspects of this disease is very low (11,12). Autoimmune diseases are one of the most widely accepted reasons for SSNHL. In this study, we examined the role of this leading cause of SSNHL through an investigation of immunological factors. 

## Materials and Methods

After approval by the ethics committee and after obtaining informed consent, this prospective case-controlled study was performed in patients who referred to the ear, nose, and throat (ENT) ward with SSNHL (inclusion criteria). Participants with acute inflammation, infection, acute or chronic renal failure, chronic renal disease, acute or chronic pulmonary thrombosis, coronary artery disease, inflammatory bowel disease, history of otologic surgery, history of ear or head trauma, hematologic disease, and history of any viral diseases were excluded.Fifty-six patients with SSNHLoccurring within a 72-hr period were selected as the case group and 56 participants who had no recent history of disease were assigned to the control group. Audiometric measurements consisted of pure tone audiometry (PTA), audiometric speech, including Speech Discrimination Score (SDS)/Speech Reception Threshold (SRT), and immittance variables, including tympanometry and stapes reflexes. All tests were performed using a similar audiometer and one speech.

In addition, blood samples containing ethylenediaminetetraacetic acid (EDTA) were taken from all participants. Next, mean values of immunological factors including erythrocyte sedimentation rate (ESR), C-reactive protein (CRP), rheumatoid factor (RF), antinuclear antibody (ANA), anti-neutrophil cytoplasmic antibody (ANCA) (C,P), anti-cyclic citrullinated peptide (anti-CCP), DSM, hemoglobin (Hb), complement component 3 (C3),and complement component 4 (C4) were obtained in each blood sample. To reduce bias, all tests were carried out by one laboratory. All data were registered using a form, and analyzed using SPSS (version 20) and running statistics, the Chi-square test, and the t-test. To explain the relationship between the SSNHL and the defined variables, Spearman and Pearson correlation tests were applied. P<0.05 was considered as the significant level.

## Results

There were 31 (55.4%) male and 25 (67.64%) female participants in the control group; whereas in the case group, 26 (46.4%) and 30 (53.6%) participants were male and female, respectively (P=0.54).The mean age of participants was 36.2 ± 13.4 years in the control group and 40.80 ± 13.37 years in the case group. The two groups were homogeneous with respect to age.

Mean ESR was 9.54 ± 6.66 in the control group and 16.12 ± 16.83 in the case group, and the results revealed a statistically significant difference between two groups in this regard (P=0.007). With respect to CRP, 100% of participants in the control group, 89.3% in the case group, and 96.96% of the participants in total, had negative CRP. The results of the Chi-square test showed a lack of association between CRP positivity and a higher incidence of SSNHL (P=0.29). All of the control group, 92.9% of the case group, and 95.5% of all participants had a negative RF. There was no significant correlation between the incidence of SSNHL and positive RF according to results of the Chi-square test (P=0.24). The two groups were also compared in terms of ANA factor.Independent t-test results demonstrated higher ANA levels in the case group compared with the control group, which resulted in an increase in SSNHL (P=0.01). Mean anti-CCP was 14.67 ± 65.09 in the control group and 3.23 ± 2.9 in the case group. An independent t-test showed that anti-CCP elevation did not significantly increase the incidence of SSNHL (P=0.35). Mean anti-DS DNA was 0.44 ± 0.28 in the control group and 0.54 ± 0.58 in the case group. An independent t-test showed that the anti-Ds DNA increment did not significantly increase the incidence of SSNHL (P=0.37). 

Mean values of Hb were also compared in the two groups. Overall, the results revealed lower Hb (not anemia) in the case group, indicating that hemoglobin decline, even in non-anemia cases, may be an important determinant of susceptibility (P=0.3). The difference between the two groups was statistically significant with respect to mean C3 factor (155.14 ± 49.52 in the control group vs. 126.29 ± 31.53 in the case group; P=0.001) and C4 factor (26.22 ± 11.01 in the control group vs. 36.15 ± 8.98 in the case group; P=0.001). It can be concluded that a C4 level below the normal range increases the incidence of SSNHL (P=0.001), while C3 elevation significantly increases the incidence of SSNHL (P<0.001). 

Mean white blood cell (WBC) level was 8.79 ± 3.24 in the control group and 8.72 ± 3.80 in the case group. There was no significant difference between the two groups in terms of WBC (P=0.49). Mean neutrophil factor in the control group was 59.77±11.87 and in the case group was 59.53±12.84, with no significant difference between the two groups (P=0.95). The two groups were not statistically different in terms of lymphocyte factor (37.55 ± 12.55 in the control group vs. 37.33 ± 11.54 in the case group; P=0.88), eosinophil factor (1.41 ± 1.97 in the control group vs. 1.28 ± 1.36 in the case group; P=0.82), or platelet factor (236.21 ± 77.10 in the control group vs. 257.04 ± 81.55 in the case group; P=0.39). 

Monocyte factor was 1.7 ± 1.40 in the control group and 1.41 ±1.57 in the case group (P=0.02). 

The results of an independent t-test showed that mean WBC, neutrophils, lymphocytes, eosinophils, and platelets were not significantly different between the two groups. However, mean monocytes were higher in the case group, indicating that monocyte elevation significantly increases the prevalence of SSNHL (P=0.02).

## Discussion

This study aimed to investigate the immunological factors in patients with SSNHL. The study was conducted in 56 individuals who had no history of infectious or systemic disease and 56 patients with SSNHL. The results of this investigation indicated a relative relationship between SSNHL and patients’ immunological factors. 

Various studies have been conducted on the association between immunity and hearing loss. For example, Süslü et al investigated the role of ESR, an immune factor, in reducing SSNHL. The Süslü study reported a significant relationship between these two variables (13), consistent with the result of this study and supporting the relationship between ESR changes and SSNHL. However, Chang revealed no significant association between the aforementioned variables (14).

This discrepancy can be resolved by performing further studies and obtaining more detailed and accurate results. A few studies have been conducted on the relationship between CRP and the incidence of SSNHL. For instance, Göde et al reported a significant relationship between CRP serum level and SSNHL, and showed that CRP had a greater effect on low-frequency SSNHL (15). According to Masuda et al, CRP serum level is significantly associated with a decrease in SSNHL; however, they concluded that CRPhas a greater effect on*high-frequency*SSNHL (16). The results of the current investigation, in contrast, revealed that CRP affects neither low-frequency nor*high-frequency* SSNHL. *These*discrepancies may be due to certain underlying diseases *hidden*inplain*sight* or to different laboratory conditions. Overcoming these controversies needs further research. 

In line with similar studies, RF was also examined in this study. For example, Rajati et al evaluated 83 patients with hearing loss and found that six patients had a high RF titer. They concluded that the titer level of RF cannot be used to determine the cause of SSNHL, and that these patients are likely to have subtle changes in their RF serum levels (17). Kastanioudakis et al explored the effects of RF on the development of SSNHL and noted that patients with high RF titer may suffer from approximately 35% *inner ear damage*. Accordingly, they concluded that RF has no correlation with SSNHL (18).With respect to the results of related studies and the present study, it cannot be assumed that RF is an effective factor in the development of SSNHL, and further studies are suggested. 

ANA has been studied as an important immunological factor in various studies, including the present study. Di Leo et al identified a significant relationship between ANA and inner ear damage. According to Di Leo et al, inner ear damage induced by high serum levels of ANA may reduce the effects of corticosteroids on the damaged tissue, leading to damage progress (19). In another study by Süslü et al, ANA was not evaluated as a factor in the development or treatment of SSNHL; however, they suggested further investigations to determine the role of ANA in SSNHL diagnosis and treatment (13). The present investigation evaluated the role of ANA in SSNHL patients; however, no correlation was observed between ANA and the development or treatment of SSNHL. The contradictions in these studies can be attributed to the small number of participants or the different conditions under which the studies were conducted. 

The role of anti-CCP in the development of SSNHL was investigated and confirmed by a few studies. Lobo et al identified the increasing effect of this factor on patients with rheumatoid arthritis who also suffered from SSNHL (20). This, however, is in contrast with the current study revealing no significant effect for anti-CCP in this regard. Due to the lack of reliable studies in this field, no comprehensive and accurate conclusion can be drawn, and further studies are needed. The role of anti-Ds DNA in SSNHL was explored for the first time in this study, but no significant association was observed. Future studies are recommended to evaluate the correlation of this factor with SSNHL development and treatment. 

A large number of studies examined the association between anemia and SSNHL. For instance, Schieffer evaluated 20,133 individuals in a cohort study and found that the risk of developing SSNHL is high in children and adults suffering from iron deficiency (21). Almuha was et al discovered an association between sickle cell anemia and SSNHL, and recommended future investigations to provide detailed information to prevent SSNHL in this group of patients (22). In the present study, the mean Hb in both groups was in the normal range, but a lower Hb level was reported in the case group. In this regard, no generalization can be made due to these discrepancies, and more precise investigations are suggested. 

C3 and C4 factors play an important role in the immunology of the body, and we found a significant correlation between these two factors and SSNHL development. However, Cho et al found no significant correlation between these two factors and the initiation of this disease (23). Both Andonopoulos and Rajati concluded that in patients with a rheumatic disease, these factors can contribute to inner ear involvement leading to poor patient recovery (17,24). This discrepancy can be attributed to the fact that their patients were treated with corticosteroids due to their underlying rheumatoid arthritis, while our patients did not receive corticosteroids. Monocytes are immune cells that have a major role in inducing inflammation in patients. This study investigated this relationship for the first time, and showed that the inflammatory process increases monocyte count. Further studies are recommended in this regard.

## Conclusion

According to the results of this study, since some of immunological factors were significantly higher in patients with SSNHL, it can be concluded that there is an association between these immunological factors and SSNHL. Further studies are recommended to accurately determine the effect of these factors on the development of SSNHL and its treatment.
